# A bibliometric and visualization analysis of global research status and frontiers on autophagy in cardiomyopathies from 2004 to 2023: a correspondence

**DOI:** 10.1097/MS9.0000000000002594

**Published:** 2024-09-25

**Authors:** Cui-feng Ji, Jian Gan, Yan-dong Miao

**Affiliations:** a Electrocardiogram Room, Yantai Affiliated Hospital of Binzhou Medical University, The 2nd Medical College of Binzhou Medical University; b Department of Gastroenterology, Yantai Affiliated Hospital of Binzhou Medical University, The 2nd Medical College of Binzhou Medical University; c Cancer Center, Yantai Affiliated Hospital of Binzhou Medical University, The 2nd Medical College of Binzhou Medical University, Yantai, Shandong, People’s Republic of China

*Dear Editor*,

In our recent scholarly investigation, we meticulously reviewed the insightful study by Zeng *et al*., titled ‘A bibliometric and visualization analysis of global research status and frontiers on autophagy in cardiomyopathies from 2004 to 2023’^1^. This study elucidated numerous potential connections between autophagy and cardiomyopathy; it provides valuable references for understanding the current status, research hotspots, and future trends in the field of autophagy in cardiomyopathy, offering guidance and insights for researchers and clinicians. Nonetheless, it behooves us to present some suggested improvements to the methodologies utilized for information retrieval in their study, alongside some additional methodological supplements.

Bibliometrics was first introduced at the beginning of the 20th century and formed an independent discipline around the 1970s. Now, bibliometrics has become widely applied in literature analysis. Bibliometrics analyzes and processes detailed information such as authors, keywords, journals, countries, institutions, and references. Thus, it provides a quantitative method for reviewing and investigating, and it can be used to predict the development direction of a field. As we all know, precision in formulating search strategies is crucial in bibliometric analysis. The authors indicated that their primary data was sourced from the Web of Science Core Collection (WoSCC), which comprises an array of several sub-databases such as the Science Citation Index Expanded (SCI-EXPANDED), Social Sciences Citation Index (SSCI), Arts and Humanities Citation Index (AHCI), Conference Proceedings Citation Index-Science (CPCI-S), and so on. Previous studies have suggested that incorporating all these sub-databases might not be optimal for identifying relevant articles^2^ and amalgamating a heterogeneous range of databases in a single bibliometric analysis may not be judicious. Therefore, the SCI-EXPANDED is frequently regarded as the most suitable for such studies^3^. Consequently, it is essential for researchers to explicitly specify the databases utilized to enhance the transparency and replicability of their data retrieval process.

In their study, Zeng *et al*. reference the procurement of original data from the WoSCC; it should be noted that the author should emphasize that SCI-EXPANDED of WoSCC the original data comes from. Furthermore, narrowly defined search scope may inadvertently omit pivotal studies, affecting the analysis’s integrity. He *et al*.’s^4^ approach contrasts with more expansive strategies, such as using the incorporation of wildcard characters, such as ‘*’, facilitates the inclusion of various terminological derivatives, for example, enabling ‘Cardiomyopath*’ to yield results for ‘Cardiomyopathy,’ ‘Cardiomyopathies,’ and related terms. In addition, although the author’s search strategies are based on Medical Subject Headings (MeSH), it cannot be denied that some expressions for specific diseases are not included in the search strategies. Taking cardiomyopathy as an example, other expressions include Cardiac storage disease, Cardiac storage disorder, Cardiac storage disease, etc., which are not included in MeSH related to cardiomyopathy. Our recommended search methodology was outlined in Supplementary Table S1 (Supplemental Digital Content 1, http://links.lww.com/MS9/A607). This approach, incorporating, aims to capture a more comprehensive literature spectrum.

In our refined search, conducted from 1 January 2004 to 31 December 2023, and completed on 18 July 2024, we identified a total of 4117 records. After meticulously excluding process by C.f.J. and J.G., in instances of disagreement, Y.-d.M., was summoned to adjudicate and ensure consensus. We finalized a selection of 3485 English articles for analysis, consisting of 2697 research articles and 788 review articles. Which emanate from 746 distinct journals, authored by 14 503 scholars, and have exhibited an annual growth rate of 23.9%. A comprehensive visualization analysis of these publications is provided in Figure 1A. The literature on the research field demonstrates a significant upward trend over the past 20 years, peaking in 2023 with 479 articles (Fig. 1B). The number of papers published by various countries, China has contributed over 2000 articles, followed by the USA, Japan, Italy, Canada, and others (Fig. 1C, D). Within this research milieu, the number of publications by multiple universities in China ranks among the top (Fig. 1E).

**Figure 1:**
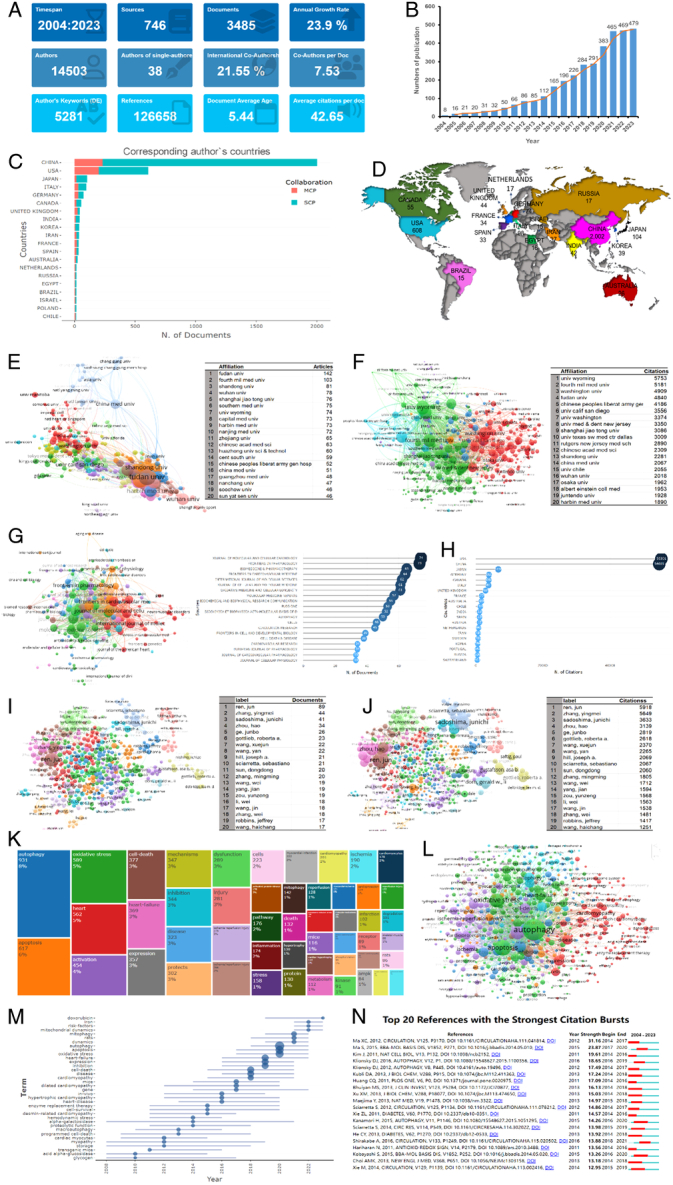
A comprehensive bibliometric analysis of autophagy in cardiomyopathies. (A) Overview of autophagy in cardiomyopathies, utilizing the ‘Bibiometrix’ package. (B) The trajectory of annual publications over the past 20 years. (C) The author’s countries. MCP, multiple country publications; SCP, single country publications. (D) Overview of the global map of scientific productivity. (E) A VOSviewer analysis of the networks of institutions and the top 20 institutions about articles published. (F) A VOSviewer analysis of the networks of institutions and the top 20 institutions about citations. (G) A VOSviewer analysis of the networks of journals and the top 20 sources of articles published. (H) The national citation ranking. (I) A VOSviewer analysis of the networks of authors and the top 20 authors of articles published. (J) A VOSviewer analysis of the networks of authors and the top 20 authors about citations. (K) TreeMap of the frequency of keywords within the scope of autophagy and cardiomyopathies. (L) A VOSviewer analysis of the networks of keywords. (M) The dynamic process of trend topics in the field of autophagy and cardiomyopathies during 2004–2023. (N) Top 20 references with the strongest citation bursts in the field of autophagy and cardiomyopathies.

In terms of source citation analysis, the University of Wyoming leads with 5753 citations (Fig. 1F). ‘Journal of Molecular and Cellular Cardiology’ and ‘Frontiers in Pharmacology’ have been identified as the outstanding journals in this field, with 74 and 73 articles published, respectively (Fig. 1G). In the national citation ranking, USA is the most cited country, ranking first (Fig. 1H). Ren, Jun is distinguished as the most prolific author and also the most cited author (Fig. 1I, J). Keywords related to the field of cell death account for a considerable proportion (Fig. 1K, L). Figure 1M illustrates the trend topics from the year 2004 to 2023. Figure 1N showcases the top 20 references with the strongest citation bursts.

Compared to the results of Zeng *et al*., our study identified 3485 articles, whereas theirs found 2279 articles. It is crucial to highlight that substantial variations in publication numbers can significantly impact various quantitative metrics. These metrics include publication counts, leading countries, institutions, citation numbers, authors, journals, keywords, and references. Such fluctuations underscore the critical importance of meticulously crafting an appropriate retrieval formula, which forms the bedrock of any objective bibliometric analysis.

In summary, while acknowledging the contributions of Zeng *et al*., we posit that our refined methodology offers relative precision and accuracy in analyzing study trends related to autophagy in cardiomyopathies. Our findings build upon their work with great value and could be a supplement to their work. This multi-tool approach not only enriches the data sources but also enhances the reliability and depth of the analysis results, providing researchers with more comprehensive references and guidance.

## Ethical approval

Not applicable.

## Consent

Not applicable.

## Source of funding

This work was supported by Shandong Provincial Natural Science Foundation (ZR2024QH658), Shandong Province Medical and Health Science and Technology Development Plan Project (No. 202303031093) and Science and Technology Program of Yantai Affiliated Hospital of Binzhou Medical University (No. YTFY2022KYQD07).

## Author contribution

C.-f.J.: conceptualization, formal analysis, literature search, methodology, software, writing – original draft; J.G. and Y.-d.M.: supervise, conceptualization, funding acquisition, formal analysis, methodology, software, writing – review and editing. All authors reviewed the manuscript.

## Conflicts of interest disclosure

The authors declare no conflicts of interest.

## Research registration unique identifying number (UIN)

Not applicable.

## Guarantor

Yan-dong Miao.

## Data availability statement

The raw data underpinning the conclusions of this article will be made accessible by the authors without undue reservation. For further inquiries, please contact the corresponding author.

## Provenance and peer review

Not applicable.

## Supplementary Material
