# Supplementary material for: A bibliometric and visualization analysis of global research status and frontiers on autophagy in cardiomyopathies from 2004 to 2023: a correspondence
Source: Ann Med Surg (Lond). 2024 Sep 25;86(11):6893–5. doi: 10.1097/MS9.0000000000002594 (PMC11543204; doi:10.1097/MS9.0000000000002594)
Supplement: Supplementary file 1 [file ms9-86-6893-s001.docx]

**Table S1:** Retrieval strategy of autophagy in cardiomyopathies

|  | Search Criteria | Records |
| --- | --- | --- |
| Records identified through the WoSCC database  searching  (SCI-EXPENDED) | #1: (TS=(Cardiomyopath* OR Myocardiopath* OR Myocardial disease* OR Myocarditis* OR Myocardial injury* OR Myocardial damage* OR Impaired myocardium* OR Myocardial involvement* OR Cardiac danon disease* OR Cardiac storage disorder* OR Cardiac storage disease* OR Secondary* cardiomyopath* OR Secondary* myocardial disease* OR Primary* cardiomyopath* OR Primary* myocardial disease* OR Alcoholic* cardiomyopath* OR Alcoholic* myocardiopath* OR Dilated* cardiomyopath* OR Dilated* myocardiopath* OR Hypertrophic* cardiomyopath* OR Hypertrophic* myocardiopath* OR Restrictive* cardiomyopath* OR Chagas cardiomyopathy* OR Diabetic cardiomyopath* OR Endocardial fibroelastosis* OR Isolated noncompaction of the ventricular myocardium* OR Endomyocardial fibrosis*))  #2: (TS=(Autophag* OR Macroautophagy* OR Microautophagy* OR Autophagosome* OR Lysosome* OR Autophagic flux* OR Chaperone* autophag* OR Mitophagy* OR Lipophagy* OR Ferritinophagy* OR Aggrephagy* OR Clockophagy* OR Nucleophagy* OR Xenophagy* OR Autophagocytosis* OR Reticulophagy* OR Ribophagy* OR ER phagy* OR Self-eating* OR LC3*)) | 280,236  127,625 |
|  |  |  |
| Time:2004-01-01–2023.12.31  Languages: English | # 3= #1 AND #2 | 4,117  4,093 |
| Excluded Literature | Meeting abstract, News, Briefing paper, Letters, duplications, and irrelevant. | 608 |
| Remaining Publications | Article (n=2,697), Review article (n=788) | 3,485 |
